# Preparation process of mesophase pitch-based carbon fiber: a review

**DOI:** 10.1039/d5ra05325k

**Published:** 2025-10-20

**Authors:** Mingzhi Wang, Saif Ullah, Muhammad Rizwan, Xiaolong Zhou

**Affiliations:** a International Joint Research Center of Green Energy Chemical Engineering, East China University of Science and Technology China 1365355476@qq.com Xiaolong@ecust.edu.cn

## Abstract

To investigate the preparation process of high-performance pitch-based carbon fibers, researchers have systematically analyzed the key stages involved, including the characterization of mesophase pitch, melt spinning, stabilization (pre-oxidation), carbonization, and graphitization. This paper provides a comparative evaluation of the advantages and disadvantages of each process step, discusses strategies for process optimization, highlights critical operational considerations, and examines improvements in experimental equipment. In addition, targeted optimization strategies are proposed. The comprehensive analysis presented herein offers a complete experimental framework that enhances efficiency, reduces costs, improves safety, and ultimately boosts the performance of the final carbon fiber products. This work aims to serve as a valuable theoretical reference for researchers in related fields.

## Abstract 2

This paper provides a comparative evaluation of the advantages and disadvantages of each process step for the preparation of pitch-based carbon fibers, discusses strategies for process optimization, highlights critical operational considerations, and examines improvements in experimental equipment.

## Graphical abstract

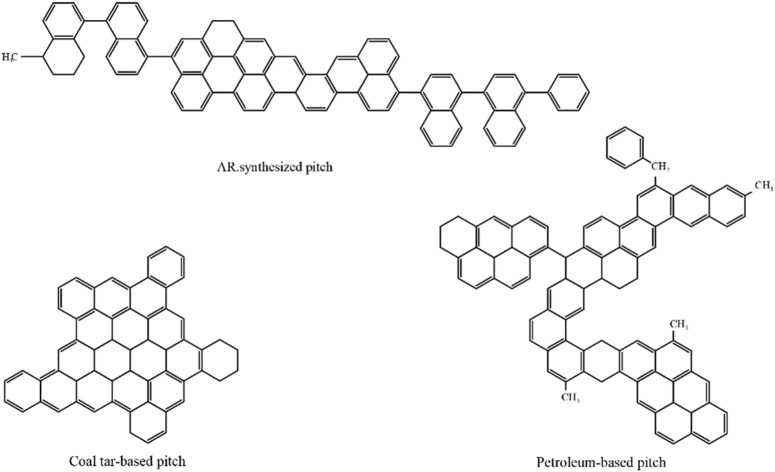

## 1. Introduction

Pitch-based carbon fibers have found widespread applications across various fields.^1,2^ The main types of carbon fibers include PAN-based carbon fibers and pitch-based carbon fibers, with the latter further divided into isotropic pitch-based and anisotropic pitch-based carbon fibers (*i.e.*, mesophase pitch-based carbon fibers). Polyacrylonitrile (PAN)-based carbon fibers are produced from polyacrylonitrile precursors through stabilization, carbonization, and subsequent graphitization processes. In contrast, isotropic pitch-based carbon fibers are derived from isotropic pitch *via* melt spinning, followed by infusibilization and subsequent carbonization treatments.^3^ Compared with PAN-based and isotropic pitch-based carbon fibers,^4,5^ mesophase pitch-based carbon fibers exhibit superior tensile modulus, electrical conductivity, and thermal conductivity. The core advantages of mesophase pitch-based carbon fibers lie in their exceptionally high thermal conductivity and modulus. Their thermal conductivity far exceeds that of PAN-based carbon fibers, making them ideal materials for thermal management and heat dissipation. At the same time, they possess a higher elastic modulus, exhibiting outstanding resistance to deformation. Moreover, after high-temperature graphitization, they display excellent electrical conductivity and typically feature a negative coefficient of thermal expansion. These typical carbon fibers have been applied in the fields of intelligence and self-healing composites.^6–9^ Table S1 is the market share of carbon fiber within the current time frame. Additionally, they offer high carbon yield and are more amenable to graphitization, making them highly applicable in sectors such as construction, aerospace, healthcare, and defense. However, the China's technology in this area remains underdeveloped compared to the advanced carbon fiber production technologies in countries like Japan and the United States.^10^ Despite technological limitations, mesophase pitch-based carbon fibers have the advantage of significantly lower production costs,and benefit from an abundant or even excessive supply of raw materials, suggesting promising prospects for future development.^11–13^

With the rapid advancements in quality of life and science and technology, mesophase pitch-based carbon fibers have increasingly permeated various aspects of both industry and daily life.^14,15^ Nonetheless, carbon fibers produced domestically face numerous challenges. One major issue lies in the relatively mediocre performance of current mesophase pitch-based fibers and the lack of well-established design and process experience.^16^ While general-purpose pitch-based carbon fibers have found stable applications, their usage remains limited, highlighting the greater importance of advancing high-performance mesophase pitch-based variants.^17–19^ Chinese universities and research institutes have made significant contributions to the modification and development of pitch-based carbon fibers.^20–23^ Enhancing the performance of mesophase pitch-based carbon fibers involves not only process optimization but also improving the molecular structure and composition of the raw mesophase pitch itself.^24,25^

The performance of high-quality mesophase pitch-based carbon fibers is fundamentally influenced by the molecular composition and structural characteristics of the mesophase pitch precursor. Therefore, understanding the correlation between the properties of mesophase pitch and the structure and performance of the resulting carbon fibers is of considerable significance.^26–28^ Most of the current review articles in this field tend to summarize and analyze a specific process, with different sections often lacking logical coherence and continuity. Based on recent literature, this paper outlines a comprehensive preparation process for mesophase pitch-based carbon fibers—from raw mesophase pitch through melt spinning, stabilization, carbonization, and graphitization—offering valuable insights and practical guidance for researchers in the field.

## 2. Mesophase pitch

**Fig. 1:**
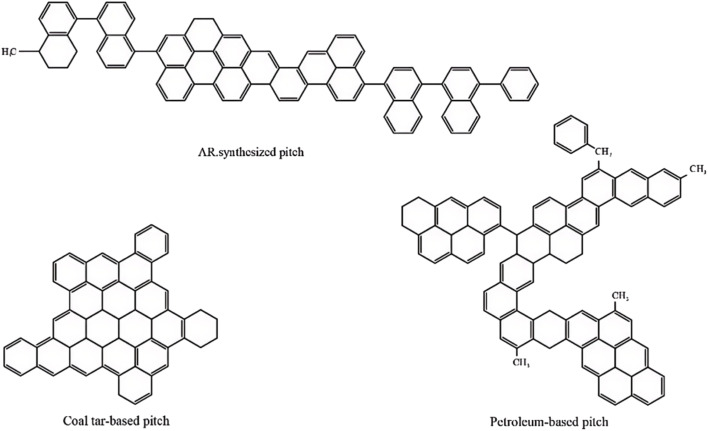
Molecular structure models of mesophase pitch in various precursors.

Mesophase pitch (MP) is primarily composed of polycyclic aromatic hydrocarbons. It is typically derived from petroleum fractions, coal tar derivatives, or various types of asphalt through thermal polycondensation. Alternatively, it can also be synthesized by catalytic condensation of high-purity aromatic compounds.^29–31^ The representative molecular structure is illustrated in Fig. 1.

**Fig. 2:**
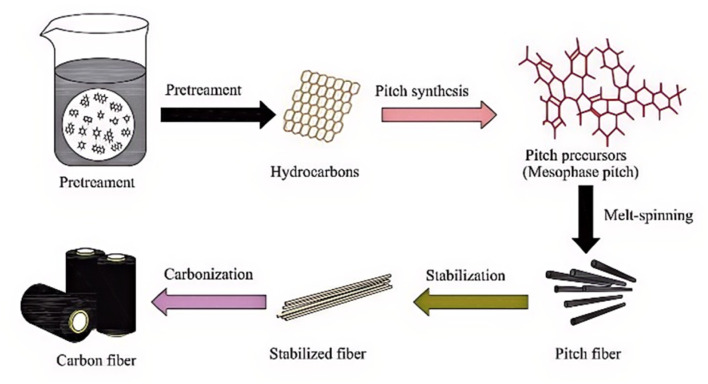
Schematic diagram of the process for preparing pitch-based carbon fibers from mesophase pitch.

As a critical precursor for numerous high-performance carbon materials, mesophase pitch can be used to fabricate carbon electrodes, needle coke, carbon foam, and carbon fibers. After undergoing a melting process, mesophase pitch is spun into fibers with molecules oriented along the fiber axis. These precursor fibers are then carbonized and graphitized to form graphite microcrystals,^32–35^ as shown in the schematic in Fig. 2.

The degree of molecular orientation during spinning plays a decisive role in determining the comprehensive performance of the final carbon fiber. Therefore, understanding the fundamental characteristics of mesophase pitch and optimizing the spinning process are essential steps toward the preparation of high-performance mesophase pitch and corresponding pitch-based carbon fibers. The following sections summarize analytical methods for evaluating MP,^36–59^ based on recent research.

### 2.1 Roles of various characterization methods for mesophase pitch

#### 2.1.1 Elemental analysis of MP

The atomic hydrogen-to-carbon (H/C) ratio of mesophase pitch derived from petroleum-based raw materials generally falls within the range of 0.5 to 0.8.^36–38^ In contrast, MP synthesized directly from coal-based precursors tends to have even lower H/C ratios. These types of pitch often contain high levels of aromatic and condensed-ring structures, along with small amounts of alkyl substituents.

By appropriately balancing aromatic content and softening point, it is possible to produce spinnable mesophase pitch that has both a high mesophase content and a relatively low softening point. During this process, it is crucial to minimize the content of heteroatoms such as nitrogen (N), oxygen (O), and sulfur (S), as these elements tend to form small gas molecules during subsequent heat treatments, which can severely disrupt the internal structure of the fibers and degrade the mechanical properties of the resulting carbon fibers.

Furthermore, controlling the ash content in mesophase pitch is essential. For pitch to be considered spinnable, the ash content should typically be kept below 70 ppm to ensure product quality and stability.

#### 2.1.2 Group composition analysis of mesophase pitch

The performance of mesophase pitch can be inferred by analyzing the proportion of its group components.^39–41^ Among them, *n*-heptane-soluble (HS) and toluene-soluble (TS) fractions are considered light components. These lighter fractions can enhance the molecular fluidity of the mesophase pitch, thereby improving its spinnability. However, an excessive presence of these light components may increase the content of isotropic phases within the product, which can adversely affect the properties of the carbon materials derived from it.

Quinoline-insoluble (QI) fractions represent the heavy components in mesophase pitch. These typically have molecular weights exceeding 3000 and are difficult to decompose or melt due to their large molecular structure, making them unfavorable for the spinning process. In general, for mesophase pitch to be considered spinnable, the QI content must be controlled below 35%. When the QI content exceeds this critical threshold, the spinning performance of the pitch declines significantly.

#### 2.1.3 Fourier transform infrared (FT-IR) spectroscopy analysis of MP

The types and quantities of functional groups present in mesophase pitch can be identified using Fourier transform infrared (FT-IR) spectroscopy.^42–44^ Absorption bands near 700–900 cm^−1^, 1600 cm^−1^, and 3040 cm^−1^ are associated with the presence of aromatic compounds; stronger intensities at these wavelengths indicate higher aromatic content. Peaks at 2920 cm^−1^, 2850 cm^−1^, 1460 cm^−1^, and 1380 cm^−1^ correspond to the aliphatic components of pitch. Additionally, the absorption band at 3430 cm^−1^ reflects the hydrogen-bonded O–H groups.

By performing spectral fitting and integral analysis on the FT-IR spectra, characteristic indices such as the alkyl substitution index (*R*), *ortho*-substitution index (*I*_os_), and aromaticity index (*I*_ar_) can be calculated using empirical formulas (1)–(3). A suitable and well-balanced ratio of aromatic to aliphatic hydrocarbons is essential for producing high-performance mesophase pitch. Detailed parameters and functional group classifications are presented in Table 1.1
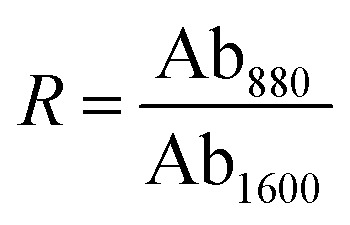
2
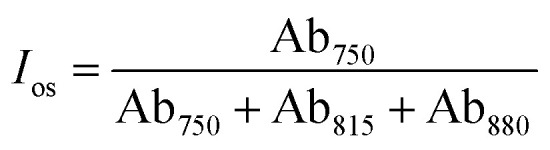
3
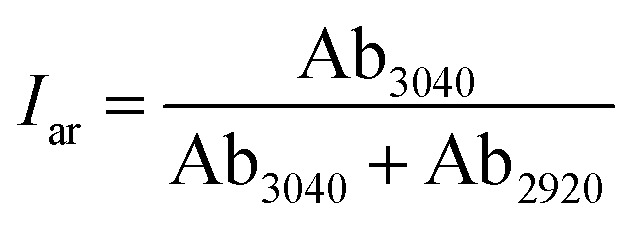


**Table 1:** Fourier transform infrared spectroscopy (FT-IR) characteristic absorption peaks of common structures

Wavenumber (cm^−l^)	Aliphatic and aromatic groups
3450–3400	–OH stretch
3050–3030	Aromatic CH stretch
2970–2850	Aliphatic CH_3_, CH_2_ and CH stretch
1775–1765	C=O stretch in ester with group attached to single-bonded oxygen
1735	C=O stretch in ester
1720–1690	C=O stretch in ketone, aldehyde, and carboxyl
1650–1630	C=O stretch, highly conjugated
1600	Aromatic C=C ring stretch, highly conjugated hydrogen-bonded C=O stretch
1510	Aromatic C=C ring stretch
1460–1440	Aliphatic chains CH_3_– and CH_2−_
1365–1355	Aliphatic chains CH_3_– or in-plane O–H bend
1275–1200	C–O–C stretch of alkyl aryl ethers, ester CC(=O)–O stretch and C–O stretch in phenols
1115–1110	C–O–C stretch of aliphatic ethers or C–O stretch in alcohols
1030	C–O stretch of aryl alkyl ethers or C–O stretch in alcohols
900–700	Aromatic CH
860	Isolated aromatic H
833 (weak)	1,4-Substituted aromatic groups
815	Isolated hand/or two neighboring H
750	1,2-Substituted, *i.e.*, four neighboring H

Among them, Ab_750_, Ab_815_, Ab_880_, Ab_1330_, Ab_1460_, Ab_1600_, Ab_2920_ and Ab_3040_ are the absorption intensity of the absorption peak at 750, 815, 880, 1330, 1460, 1600, 2920 and 3040 cm^−1^ respectively. The alkyl index (*R*), *ortho*-substitution index (*I*_os_), and aromaticity index (*I*_ar_) of mesophase pitch, by reflecting its molecular structural features, are directly associated with its processing behavior and the final properties of the resulting carbon fibers. Specifically, a higher *R* value indicates a greater number of alkyl side chains, which can lower the softening point of the pitch, improve fluidity, facilitate spinnability, and influence fiber orientation. An increase in the *I*_os_ value suggests enhanced *ortho*-substitution of aromatic rings, which may hinder molecular ordering and stacking, thereby increasing structural defects in carbon fibers and deteriorating their mechanical and thermal properties. Conversely, a higher *I*_ar_ value reflects an increased degree of aromaticity, which promotes the formation of an ordered graphite structure during carbonization, improving the modulus of carbon fibers, although compositional heterogeneity may induce fluctuations in tensile strength. Collectively, these three indices regulate the spatial conformation and aggregation state of pitch molecules, ultimately determining the microstructure and macroscopic performance of the derived carbon fibers.

#### 2.1.4 Crystalline structure analysis of mesophase pitch

##### 2.1.4.1 XRD analysis

X-ray diffraction (XRD) analysis is a widely adopted technique for characterizing the microstructure and crystalline properties of carbon materials.^45–47^ By analyzing the XRD patterns and fitting the corresponding curves, key microcrystalline parameters can be calculated using established formulas (4)–(6). In mesophase pitch, a distinct diffraction peak typically appears at approximately 2*θ* ≈ 25.5°, which corresponds to the (002) crystal plane. This peak is attributed to the hierarchical stacking of aromatic layers within the pitch during thermal treatment, reflecting the formation of layered structures derived from aromatic constituents.4
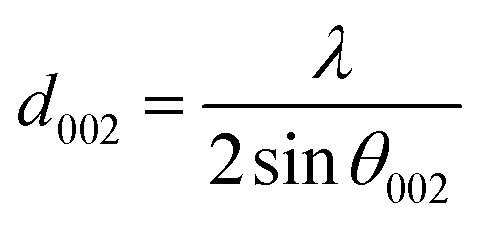
5
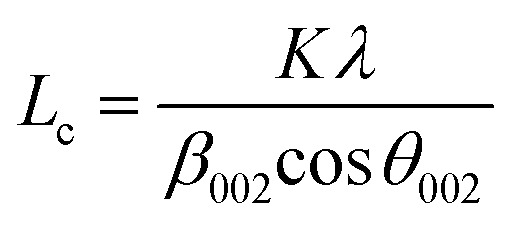
6
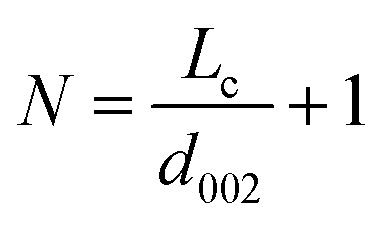
In some cases, a broad and relatively weak diffraction peak can also be observed near 2*θ* ≈ 10°, indicating the presence of a small amount of amorphous or disordered carbon structures. These features suggest that not all molecular components are graphitizable, and certain portions remain non-crystalline.

Among the commonly used XRD parameters, *d*_002_ refers to the interlayer spacing between aromatic lamellae. A smaller *d*_002_ value implies a higher degree of molecular orientation and structural compactness. Other important parameters include *L*_a_ (the lateral crystallite size), *L*_c_ (the crystallite height), and *N* (the number of stacked layers). Larger values of *L*_a_, *L*_c_, and *N* are indicative of well-developed graphite microcrystals, which are desirable for the fabrication of high-performance carbon materials with superior thermal and mechanical properties.

##### 2.1.4.2 Raman analysis

Raman spectroscopy is a widely used method for evaluating the structural order, degree of graphitization, and defect content in carbon materials.^48,49^ The analysis is primarily conducted through curve fitting of the Raman spectra, followed by the calculation of relevant structural parameters using empirical formulas. In the case of mesophase pitch, the Raman spectrum typically features two prominent peaks: the D band near 1360 cm^−1^ and the G band near 1580 cm^−1^.

The D band reflects the presence of structural imperfections, disordered regions, and non-graphitizable carbon. A larger D band area indicates a higher proportion of such disordered structures and a lower degree of molecular alignment within the pitch. In contrast, the G band arises from the in-plane vibrations of carbon–carbon bonds in sp^2^-hybridized graphitic domains. A sharper and more intense G peak is associated with a higher degree of graphitic development and structural regularity.

To quantitatively assess the structural ordering, researchers commonly use the intensity ratio of the D and G bands, denoted as *R* (*R* = *I*_D_/*I*_G_), where *I*_D_ and *I*_G_ represent the intensities of the D and G peaks, respectively. A lower *R* value corresponds to better molecular orientation and a higher degree of structural order within the mesophase pitch. This parameter serves as a key indicator for predicting the graphitization potential and final performance of the resulting carbon fiber materials.

#### 2.1.5 Polarized optical microscopy analysis of mesophase pitch

Mesophase pitch is inherently anisotropic, distinguishing it from isotropic components. Under polarized optical microscopy (POM), anisotropic regions of mesophase pitch reflect polarized light and appear bright in color, while isotropic regions tend to appear darker. This contrast allows for effective observation and analysis of the optical properties and structural features of the pitch.^50–52^ Based on recent literature, the optical textures of mesophase pitch can generally be classified into several types, including domain-type, flow-type, and mosaic-type structures. The specific classification criteria and definitions are detailed in Table 2.

**Table 2:** Evaluation criteria for optical texture

Anisotropic optical texture	Size/[μm] length	Width
Coarse flaky texture	>30	>30
Fine flaky texture	10–30	10–30
Mosaics texture	≤10	<10
Fine fiber texture	<30	<10
Coarse fiber texture	>30	>10

#### 2.1.6 Spinnability analysis of mesophase pitch

The most commonly used method for determining the spinnable temperature range of mesophase pitch is the penetration test, which offers high intuitiveness and ease of operation.^53–55^ In this method, the pitch sample is first heated to a temperature near its softening point, at which it begins to soften and allows a probe to penetrate easily. As heating continues and viscosity decreases, the pitch reaches a state where the probe can be drawn out smoothly into a fine filament with appropriate cohesion and elongation. This temperature is recorded as *T*_1_.

Upon further heating, the viscosity of the pitch may drop significantly. Once it reaches a critical point where the probe can no longer adhere to the pitch effectively or form stable filaments, the corresponding temperature is noted as *T*_2_. The range between *T*_1_ and *T*_2_ defines the optimal spinning temperature window for the mesophase pitch. This interval is crucial for evaluating the processability and thermal adaptability of the pitch during fiber formation.

#### 2.1.7 Rheological property analysis of mesophase pitch

At present, researchers in this field commonly evaluate the rheological behavior of mesophase pitch indirectly through its viscosity–temperature or viscosity–shear rate curves.^56,57^ During viscosity measurements, the apparent viscosity is typically used as a representative parameter. Mesophase pitch exhibits a negative correlation between viscosity and shear rate, indicating its nature as a non-Newtonian fluid. As the temperature increases, the viscosity of the pitch decreases significantly. Under the influence of external shear forces, the molecular orientation of the pitch structure can be improved, which enhances its spinnability. Studies have shown that mesophase pitches with better fluidity tend to yield carbon materials with a higher degree of homogeneity and structural uniformity.

#### 2.1.8 Thermal stability analysis of mesophase pitch

The thermal stability of mesophase pitch is usually assessed using thermogravimetric analysis (TG/DTG) curves.^58–60^ These analyses are typically conducted under nitrogen or oxygen atmospheres. Under nitrogen conditions, the weight loss trend of mesophase pitch is monitored to identify suitable spinning temperature ranges. Specifically, the onset of rapid mass loss is defined as the point at which the TG curve indicates a weight loss exceeding 1% per 10 °C increase. This threshold is used to help determine the upper limit of the spinning temperature, with the starting temperature often set 30–50 °C above the softening point of the pitch.

Under an oxygen atmosphere, the aliphatic chains in the mesophase pitch molecules preferentially react with oxygen. Carbon and hydrogen atoms within the pitch molecules combine with oxygen, leading to a measurable increase in sample mass. By analyzing TG/DTG curves under oxidative conditions, a significant weight gain—defined as an increase of more than 0.5% per 10 °C—is typically observed within a specific temperature range. The starting (*T*_3_) and ending (*T*_4_) temperatures of this range are recorded.

To ensure a stable and controlled pre-oxidation process during fiber production, a pre-oxidation temperature should be selected within the *T*_3_–*T*_4_ range. The selected temperature should ideally be as high as possible within this interval, while ensuring that DTG fluctuations remain minimal, so as to achieve a balance between sufficient reaction activation and structural integrity during pre-oxidation.

#### 2.1.9 Nuclear magnetic resonance analysis of mesophase pitch

Nuclear magnetic resonance (NMR) analysis of mesophase pitch primarily employs ^1^H and ^13^C spectra to precisely elucidate its complex chemical structure and molecular composition.^59–62^ This technique enables the quantitative determination of key structural parameters such as aromaticity and the degree of aromatic ring condensation. By examining the type and length of aliphatic side chains, as well as the hydrogen distribution on aromatic rings, NMR effectively distinguishes between peripheral and internal hydrogens. These insights reveal the intrinsic relationship between molecular orientation and mesophase content, thereby providing critical molecular-level evidence for optimizing spinnability and enhancing the ultimate properties of carbon fibers.

### 2.2 Chapter summary

Mesophase pitch is a complex precursor composed of various chemical components, making its spinnability difficult to assess precisely and its experimental reproducibility relatively low. Variations in reaction conditions and feedstock types can lead to significant differences in the molecular composition and structure of the resulting pitch, which in turn causes inconsistencies in the performance of the carbon fibers produced. Based on the analyses presented in Section 2.1, the following conditions must be met to prepare high-performance mesophase pitch-based carbon fibers:

(1) Optical texture: when the mesophase pitch exhibits a flow-type or domain-type texture under polarized optical microscopy, the resulting carbon fibers tend to have higher molecular alignment along the fiber axis. This promotes the formation of large, well-ordered graphite crystals, thereby improving the overall performance of the final fibers.

(2) Spinnability good spinnability is largely determined by parameters such as softening point and rheological behavior. These properties are not only dependent on the intrinsic quality of the raw materials but are also influenced by reaction parameters such as temperature, duration, and fiber winding speed.

(3) Purity it is essential to minimize the content of heteroatoms, ash, and quinoline-insoluble (QI) fractions in the mesophase pitch. These impurities can hinder the development of graphite crystallites, introducing defects into the structure, which ultimately compromise the mechanical and thermal properties of the resulting carbon fibers.

The statement that a smaller *d*_002_ value indicates higher molecular orientation and structural compactness is correct, but it can be elaborated in more detail.^30,35,36^ In the context of mesophase pitch (MP), both heat treatment and the degree of polymerization influence *d*_002_; however, not all smaller *d*_002_ values necessarily guarantee the spinnability of the fibers. Similarly, the intensity of the Raman D-band is not solely determined by the fraction of disordered regions but is also affected by domain size and curvature effects, which should be discussed in the Discussion section.

## 3. Introduction to the melt spinning process

The production of mesophase pitch-based carbon fibers typically begins with the formation of precursor fibers through melt spinning techniques. Common methods include centrifugal spinning, extrusion spinning, and melt blowing—also known as the air-blowing method—which is currently the most widely applied approach.^60–63^

In this method, mesophase pitch is first heated and converted into a molten state. Under the pressure of high-purity nitrogen gas, the molten pitch is forced through spinneret nozzles. Driven by a combination of gravity and high-velocity gas shear forces, the molten stream is extruded in filament form and collected by a winding unit, resulting in continuous and uniform filaments. The success of this spinning process has a direct impact on the cross-sectional structure and uniformity of the precursor fibers.

Studies have shown that carbon fibers with fine diameters and onion-like cross-sectional structures tend to exhibit better tensile strength, whereas fibers with radial structures demonstrate enhanced conductivity.^64^ Therefore, the melt spinning process plays a crucial role in determining the structural quality of precursor fibers and the overall performance of the final carbon fibers.

It is also worth noting that due to the liquid crystalline nature of mesophase pitch, its spinning behavior differs significantly from that of conventional polymers and is more sensitive to external factors, posing greater challenges for industrial-scale processing.^65–67^ The following sections provide a detailed analysis of the core influencing factors in the melt spinning process.

### 3.1 Core influencing factors in the melt spinning process

#### 3.1.1 Material factors—properties of spinnable pitch

##### 3.1.1.1 Mesophase content and purity

To ensure a stable and effective melt spinning process, the mesophase content of the pitch must meet stringent requirements.^68,69^ Typically, a content of over 95% is necessary to guarantee sufficient molecular orientation and favorable graphitization potential. If the mesophase content is too low, the pitch contains excessive isotropic components, resulting in a decline in the strength and modulus of the final carbon fibers.

In addition, the quinoline-insoluble (QI) content must be strictly controlled—ideally kept below 0.1 wt%. QI consists of hard particulate impurities that can easily clog the micro-nozzles of the spinneret and introduce structural defects during fiber formation, thus compromising the uniformity and mechanical integrity of the resulting fibers.

##### 3.1.1.2 Rheological properties of spinnable pitch

The viscosity of mesophase pitch is highly sensitive to both shear rate and temperature.^70,71^ When the viscosity is too low, the pitch melt lacks sufficient strength, leading to frequent resonant vibration and fiber breakage during spinning. On the other hand, excessive viscosity increases extrusion pressure, disrupts flow uniformity, and may result in spinneret blockage.

Moreover, the viscoelasticity of the pitch significantly influences behaviors such as melt swelling and melt fracture. When the elastic component is too high (the melt swelling ratio exceeds 1.5), the extrudate may expand excessively upon exiting the spinneret, destabilizing the flow field and increasing the risk of fiber fusion or surface fuzziness.

Throughout the spinning process, maintaining liquid crystalline stability is essential. A stable nematic phase must be preserved at spinning temperatures to avoid phase separation or premature solidification. These conditions are critical for achieving uniform and high-quality carbon fibers during subsequent carbonization and graphitization stages.

##### 3.1.1.3 Molecular structure, molecular weight, and distribution of spinnable pitch

The molecular weight of mesophase pitch plays a critical role in determining its viscosity, softening point, and overall spinnability.^72–74^ If the molecular weight is too high, the pitch becomes difficult to spin due to excessive viscosity; conversely, a low molecular weight may result in poor mechanical strength of the precursor fibers. A narrow molecular weight distribution is generally favorable, as it promotes stable spinning behavior and contributes to the uniformity of the resulting fibers. In contrast, a broad molecular weight distribution may cause localized variations in rheological behavior, leading to flow instability and inhomogeneities in the final carbon fiber product.

In addition, disc-like molecular structures in mesophase pitch exhibit superior stacking capability and higher molecular orientation, which enhances their graphitization potential. These structural characteristics are advantageous for achieving well-aligned carbon layers and high-performance carbon fibers during thermal treatment.

##### 3.1.1.4 Thermal stability of spinnable pitch

The melt spinning temperature window is largely determined by the softening point of the spinnable pitch.^75,76^ It is essential to ensure that the pitch maintains adequate thermal stability at the spinning temperature to prevent undesirable phenomena such as thermal oxidation, decomposition, and the formation of gas bubbles or coke particles, all of which can compromise fiber integrity.

Furthermore, it is important to avoid premature curing or crystallization of the pitch during spinning, as these processes may interfere with the proper formation and solidification of the fibers. Excessive molecular ordering during extrusion can negatively impact fiber cooling rates and hinder the development of a uniform structure, ultimately affecting the mechanical and structural properties of the carbon fiber product.

### 3.2 Process parameters affecting the melt spinning process

#### 3.2.1 Spinning temperature

Spinning temperature is the most critical process parameter and must be maintained within the nematic liquid crystalline phase range of the mesophase pitch, typically 20–50 °C above its softening point.^77,78^ If the temperature is too low, the pitch viscosity becomes excessively high, resulting in poor flowability, severe melt swelling, and a high risk of spinneret clogging. Conversely, if the temperature is too high, the liquid crystalline structure is disrupted, and anisotropic components may convert into isotropic phases. This leads to a rapid drop in viscosity, reduced melt strength, frequent fiber breakage, dripping, and a significantly increased risk of thermal degradation.

#### 3.2.2 Extrusion pressure

The extrusion pressure required to force the molten pitch through the spinneret must be stable and sufficiently high, typically exceeding 100 MPa.^79,80^ Pressure fluctuations directly impact the extrusion flow rate and fiber diameter uniformity. If the spinning pressure is too low, the flow rate may become unstable within the designated time frame, causing interruptions in the spinning process and a higher breakage rate. If the pressure is too high, the extruded fibers tend to be overly thick, which compromises the mechanical performance of the resulting carbon fibers.

#### 3.2.3 Spinning and winding speeds

The spinning rate and winding speed determine the draw ratio at the spinneret and influence the cooling and solidification behavior of the fibers during spinning.^81–83^ When the spinning rate is too low, the fibers remain in the cooling zone for too long, allowing molecular relaxation and loss of orientation. When the rate is too high, excessive tensile stress may lead to fiber breakage, and insufficient cooling can cause fiber fusion or filament coalescence. Studies have shown that increasing the winding speed can help reduce fiber diameter, and finer fibers tend to exhibit higher modulus. The production of high-modulus carbon fibers requires high spinning speeds to enable a high draw ratio and achieve a high degree of molecular alignment.

#### 3.2.4 Spinneret design and condition

The design and condition of the spinneret play a direct role in the quality and stability of the melt spinning process. Key influencing factors include:^84–86^

(1) Orifice geometry: round orifices are most commonly used, although rectangular orifices can be employed to produce flat fibers. The orifice shape affects melt shear behavior and molecular alignment.

(2) *L*/*D* ratio: a higher length-to-diameter ratio (*L*/*D* > 4) enhances shear forces during extrusion, promoting better molecular orientation and reducing melt swelling.

(3) Surface smoothness: the internal surfaces of the spinneret must be highly polished—ideally mirror-finished. Even minor scratches or burrs can lead to melt rupture or structural defects in the fibers.

(4) Cleanliness and anti-coking: the spinneret must be kept absolutely clean to avoid clogging and carbonization. Regular maintenance, such as high-temperature calcination or solvent cleaning, is recommended to maintain spinneret performance.

**Fig. 3:**
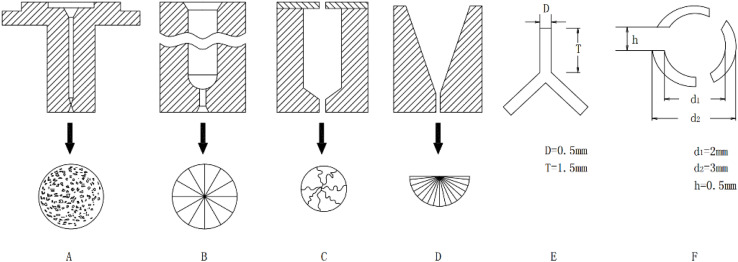
Panels (A)–(D) respectively illustrate several common microstructures of asphalt fibers, where (A), (B), (C), and (D) represent random microstructure, radial microstructure, onion-skin microstructure, and globe-like microstructure. Panels (E) and (F) demonstrate the structural parameters of commonly used spinneret orifice configurations.

Fig. 3 (ref. 86) illustrates the influence of different spinneret designs on the cross-sectional morphology of the resulting carbon fibers.

#### 3.2.5 Cooling conditions

Key experimental parameters during the cooling stage include cooling rate, cooling method, and the length and positioning of the cooling zone.^87^ In most cases, rapid cooling is applied to effectively “freeze” the highly oriented liquid crystalline structure. If the cooling rate is insufficient, it may lead to molecular relaxation or undesirable crystallization, compromising the structural integrity of the fibers.

The most commonly employed method is forced convection using cold air, where precise control of air temperature, velocity, and direction is critical to ensure uniform cooling. In some systems, water mist or contact cooling *via* chilled rollers is used to enhance cooling efficiency. Throughout the cooling process, it is essential to monitor the cooling zone length and placement carefully to ensure that solidification is completed before the filaments reach their maximum thinning point, thereby maintaining fiber uniformity and preventing defects.

#### 3.2.6 Oiling and bundling treatment

The application of a suitable amount of fiber oiling agent on the surface of the filaments serves several functions: anti-static protection, lubrication, and fiber bundling. This treatment minimizes friction and static electricity between individual filaments, thus preserving the integrity of the fiber tow during handling and winding.^88^ However, improper selection of the oiling agent or non-uniform application may negatively affect subsequent processing steps, such as stabilization or carbonization.

### 3.3 Environmental conditions for the spinning process

The entire spinning system must be protected under an inert atmosphere, typically high-purity nitrogen or argon, to strictly isolate the process from oxygen. Exposure to oxygen, especially at elevated temperatures, can result in oxidative cross-linking of the pitch, leading to charring, discoloration, or even coke formation, severely compromising fiber quality.^89,90^

In addition, cleanliness of the experimental environment is critical. Even minor particulate contamination, such as airborne dust, can adhere to the fiber surface or contaminate the molten pitch, introducing structural defects in the final carbon fiber product. Therefore, maintaining a dust-free, controlled environment is essential for achieving consistent and high-performance fiber production.

### 3.4 Optimization strategies

#### 3.4.1 Raw material optimization

Recent studies have shown that the production of high-performance carbon fibers imposes stringent requirements on the precursor materials. The selection and optimization of raw materials for mesophase pitch must address the following key aspects:^91–94^

##### 3.4.1.1 Strict raw material control

(1) Selection of raw pitch: it is recommended to use high-quality pitch with a high mesophase content. In general, the anisotropic component should exceed 95%, and the content of quinoline-insoluble (QI) substances should be kept below 0.1%, to ensure good spinnability and final fiber properties.

(2) Controlled synthesis or blending: precise tuning of the pitch preparation process is essential. Common strategies include co-carbonization and hydrogenation treatments, which help to optimize molecular weight, molecular weight distribution, rheological properties, and thermal stability—all of which are critical for stable fiber formation.

(3) Pre-use filtration: the raw pitch should undergo strict filtration before spinning. Multi-stage precision filtration is recommended, using materials such as sintered metal fiber mats or packed particle beds, to remove fine solid impurities and enhance process reliability.

##### 3.4.1.2 Efficient melt filtration

To further ensure the purity of the spinning melt and protect downstream components such as the metering pump and spinneret, high-precision and high-pressure-resistant filters should be installed in the melt delivery pipeline. A multi-stage filtration system, with progressively reduced pore sizes down to 5–10 μm, is effective for removing residual micro-particles.

Implementing automated switching and back-flushing mechanisms can ensure continuous operation of the filtration system, preventing clogging and maintaining consistent spinning conditions. This is a proven strategy to enhance the purity of the fibers, improve their mechanical performance, and extend equipment lifespan.

#### 3.4.2 Precision control and optimization of process parameters

The entire experimental process demands strict control of processing parameters to ensure fiber quality and consistency. The specific experimental variables and operational steps are outlined as follows:^95–97^

##### 3.4.2.1 Precise temperature control

(1) Zoned and multi-point temperature control should be applied to the entire melt processing system, including the melt delivery pipelines, metering pump, spinning chamber, and spinneret. Temperature fluctuations across all zones must be kept within ±1 °C to ensure thermal uniformity.

(2) Instruments such as differential scanning calorimetry (DSC) and rheometers should be used to determine the optimal spinning temperature range. Within this range, it is essential to identify a point where the viscosity is moderate, melt swelling is minimized, and melt elasticity is well-balanced, to ensure stable spinning behavior.

##### 3.4.2.2 Pressure stabilization

To maintain extrusion stability, melt pipeline design should be optimized to minimize dead zones and flow resistance. A high-precision gear metering pump should be used to maintain a consistent extrusion rate. When conditions allow, real-time monitoring and feedback control of extrusion pressure should be implemented to ensure smooth operation and prompt adjustment in response to fluctuations.

##### 3.4.2.3 Optimization and management of spinneret design

Spinneret design should be tailored based on the target fiber linear density, with carefully selected orifice diameter and orifice count. Experimental results have shown that a length-to-diameter (*L*/*D*) ratio of ≥4–6 provides favorable spinning outcomes. The spinneret should be constructed using high-performance materials, such as metal alloys, to ensure thermal strength, corrosion resistance, and high surface smoothness.

Regular maintenance is essential to preserve spinneret performance. This includes scheduled disassembly and inspection, high-temperature calcination, or ultrasonic cleaning with solvents to prevent clogging and maintain orifice precision.

##### 3.4.2.4 Enhanced cooling and rapid solidification

Optimizing the air-cooling system design is crucial to ensure effective fiber solidification. Key components such as air ducts and guide vanes should be configured to maintain a uniform, stable airflow field with moderate turbulence. Low-temperature, high-velocity cooling air should be used, ideally at temperatures below ambient. More efficient cooling methods are also recommended, including dual air rings, quenching rings, and water mist-assisted cooling. Additionally, the length and starting position of the cooling zone must be carefully adjusted to allow for sufficient fiber solidification at the desired location.

##### 3.4.2.5 Precise control of spinning and winding speeds

To achieve a high degree of molecular orientation in the precursor fibers, spinning speed should be maximized without causing fiber breakage. It is also important to maintain an appropriate ratio between winding speed and extrusion rate, which determines the draw ratio at the spinneret. Where possible, high-precision, low-vibration winding equipment should be employed to ensure consistent winding tension and prevent process disturbances.

##### 3.4.2.6 Optimization of oiling process

The oiling agent should be selected based on its compatibility with pitch, thermal resistance, and low volatility. Specialized oils with these properties are preferred. At the same time, the concentration, application quantity, and uniformity of the oil must be precisely controlled. Preferred application methods include roller oiling and spray oiling, both of which offer consistent and adjustable coverage to reduce fiber friction, improve bundling, and prevent electrostatic buildup.

#### 3.4.3 Environmental and system safeguards

In addition to material and process optimizations, it is essential to ensure the stability and reliability of the spinning environment and system infrastructure. The following aspects are critical:^98–100^:

##### 3.4.3.1 Comprehensive inert gas protection system

The entire melt spinning system—from the melt tank to the winding unit—should be maintained under a positive-pressure atmosphere of high-purity inert gas, typically nitrogen or argon, with the oxygen concentration kept below 10 ppm. Special attention should be given to critical interfaces, especially beneath the spinneret, where gas sealing devices should be installed to prevent oxygen ingress and ensure the thermal and structural stability of the spinning process.

##### 3.4.3.2 High-cleanliness environment

The spinning workshop should maintain a high level of cleanliness, generally meeting class 10 000 standards or higher. A clean environment reduces the risk of particulate contamination, which could otherwise adhere to the fiber surface or disrupt the melt flow, leading to defects in the final product.

##### 3.4.3.3 Advanced online monitoring and feedback control

Where conditions permit, real-time monitoring of key process parameters should be implemented. These include melt temperature and pressure, spinning tension, filament diameter, uniformity, and breakage events. By integrating these measurements with a Distributed Control System (DCS) or Supervisory Control and Data Acquisition (SCADA) system, automatic regulation and early-warning mechanisms can be established, ensuring high process stability and responsiveness.

#### 3.4.4 Goal-oriented exploration of process windows

Process window design should be tailored to the target properties of the carbon fiber.^99,100^ For example, in the preparation of high-strength, high-modulus fibers, ultra-high spinning speeds, elevated draw ratios at the spinneret, and extremely rapid cooling rates may be required to achieve maximum molecular orientation in the precursor fibers. In such cases, raw material purity and process stability must meet the most rigorous standards.

On the other hand, if the goal is to produce general-purpose carbon fibers, the process can be operated within a broader and more flexible window, placing greater emphasis on production efficiency and cost-effectiveness rather than peak performance.

### 3.5 Chapter summary

The successful implementation of melt spinning for mesophase pitch is highly dependent on the purity and tunability of the raw materials, as well as the precise control of process parameters such as temperature, pressure, spinning speed, and cooling rate. The structure of the spinneret, the stringent cleanliness of the operating environment, and the overall system integrity are equally critical. Process optimization must be approached as a systematic engineering challenge, requiring comprehensive and refined management across multiple domains, including raw material synthesis and purification, melt transport and filtration, spinning component design, thermal regulation, cooling efficiency, environmental control, and online monitoring.

The core objective is to achieve highly ordered, uniform, and stable molecular orientation of the mesophase liquid crystals during high-speed spinning, and to “freeze” this orientation to produce near-defect-free pitch-based precursor fibers. These optimized precursors provide a robust foundation for subsequent stabilization, carbonization, and graphitization, ultimately enabling the fabrication of high-performance carbon fibers.

## 4. Introduction to stabilization (pre-oxidation) treatment

Stabilization treatment,^101–103^ also referred to as non-melting treatment, is a crucial intermediate step before carbonization. Mesophase pitch is inherently thermoplastic; therefore, if exposed directly to high temperatures during thermal treatment, the surface of the fibers may soften or even fuse together, severely compromising their mechanical integrity and structural uniformity.

**Fig. 4:**
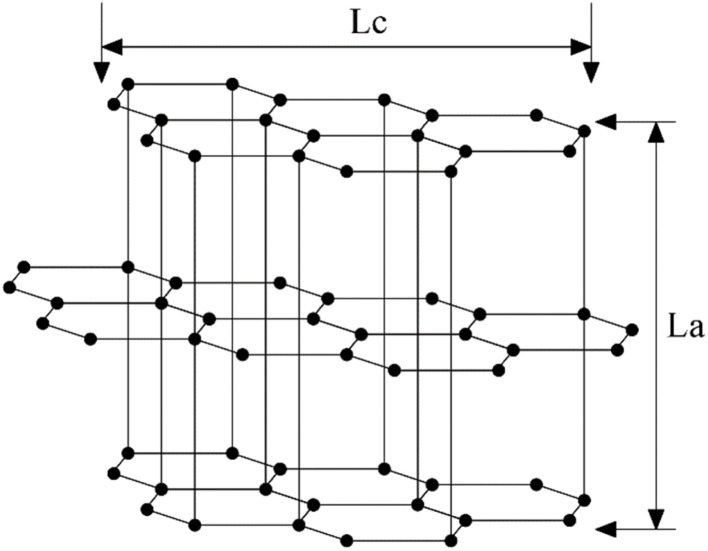
Standard graphite microcrystalline structure.

To ensure the thermal processing of precursor fibers proceeds in a stable manner, chemical crosslinking of the pitch molecules is required. The goal is to convert the thermoplastic structure into a thermosetting network by forming larger carbon-based frameworks, thereby enhancing the thermal resistance of the fibers. This transformation is achieved through oxidative reactions that convert reactive groups such as methyl and methylene into carbonyl, carboxyl, and ether bonds. These new functional groups significantly improve the thermal stability of the fibers, promote axial molecular alignment, and enhance tensile strength. Furthermore, they contribute to the development of more well-defined graphitic microcrystals,^104^ as illustrated in Fig. 4.

Currently, stabilization treatments are broadly classified into gas-phase and liquid-phase oxidation methods.^105,106^ Gas-phase stabilization involves the use of oxidizing atmospheres such as oxygen, ozone, air, carbon dioxide, and sulfur dioxide. Liquid-phase methods employ strong oxidizing agents such as potassium permanganate, hydrogen peroxide, and sulfuric acid. Among these, air oxidation is the most widely used due to its low cost and minimal environmental impact.

The effectiveness of mesophase pitch stabilization is influenced by a wide range of factors, including material properties, processing parameters, and environmental conditions. The following section will analyze the key influencing factors and their mechanisms of action in detail.

### 4.1 Influence of raw material properties on stabilization (pre-oxidation) treatment

#### 4.1.1 Raw material characteristics

The mesophase pitch used for stabilization treatment must meet strict performance requirements. Several key factors must be carefully controlled to ensure effective pre-oxidation:^107–110^

##### 4.1.1.1 Composition and structure of mesophase pitch

A critical parameter is the content of quinoline-insoluble (QI) substances. Excessively high QI levels can lead to non-uniform oxidation, whereas levels that are too low may result in reduced crosslinking efficiency. The molecular weight distribution should also be monitored: a narrow distribution facilitates uniform oxidation, while a broad distribution may cause local over-oxidation or under-reaction.

The rheological behavior of the pitch is another key consideration. Pitch with a high softening point requires higher oxidation temperatures but must avoid deformation or melting during treatment. Furthermore, impurities—especially ash or metallic contaminants such as Na or K—should be minimized, as they may catalyze side reactions and alter the intended oxidation pathways.

##### 4.1.1.2 Advantages and disadvantages of mesophase content and morphology

When the mesophase content is high, the pitch exhibits higher reactivity due to the abundance of polycondensed aromatic structures and greater molecular orientation. These features promote faster oxidation reactions, which may lead to localized overheating or non-uniform crosslinking. High mesophase content also favors dense crosslink formation, generating structures such as carbonyls and ether bonds that improve thermal stability. However, excessive crosslinking can increase the brittleness of the fibers.

Another drawback is the narrower process window: higher reactivity demands precise control of oxidation temperature and time. If the surface oxidizes too rapidly while the interior remains under-treated, a “skin-core structure” may develop, compromising fiber integrity.

Conversely, when the mesophase content is low, the pitch contains more isotropic components, leading to slower and milder oxidation reactions. Although this extends the oxidation time, it often results in greater uniformity. However, the mechanical properties of the final carbon fiber may be limited, as lower molecular orientation and graphitization degrees reduce modulus and tensile strength.

#### 4.1.2 Mechanisms by which raw materials influence stabilization

The impact of mesophase pitch on the stabilization process arises from several underlying mechanisms:^111–113^

(1) Oxygen diffusion rate: high mesophase content can cause rapid surface crosslinking, which inhibits oxygen penetration into the fiber core. In such cases, a temperature gradient strategy, such as stepwise heating, is necessary to ensure uniform oxidation throughout the fiber.

(2) Volatile release: mesophase pitch generally produces fewer volatiles during pyrolysis. However, when isotropic content is higher (as in low-mesophase pitch), more low-molecular-weight gases may be released, increasing the risk of porosity in the fiber. To counteract this, gas flow rates should be adjusted to aid volatile removal and prevent structural defects.

### 4.2 Influence of process parameters on stabilization (pre-oxidation) treatment

The effectiveness of the pre-oxidation process is significantly influenced by several key processing parameters, each playing a distinct role in determining fiber structure and final performance. The primary influencing factors are outlined as follows:^114–116^

#### 4.2.1 Influence of temperature

Pre-oxidation typically begins within the temperature range of 200–250 °C, initiating molecular oxidation reactions. However, when the temperature exceeds a critical threshold, softening and deformation of the pitch-based fibers may occur. The heating rate is usually set between 1–5 °C min^−1^. A faster heating rate can result in large internal–external temperature gradients, leading to the formation of “skin-core” structures, where the outer shell oxidizes faster than the core.

The final treatment temperature is generally maintained between 250–300 °C. If this temperature is set too high, the fiber structure may begin to decompose; if too low, insufficient crosslinking occurs, which significantly compromises the mechanical properties of the resulting carbon fibers.

#### 4.2.2 Influence of oxidation time

The choice of oxidation time must balance the need for sufficient molecular crosslinking with process efficiency. If the oxidation time is too short (less than 30 minutes), the fibers may remain incompletely stabilized, resulting in poor structural integrity during subsequent carbonization. Conversely, if the oxidation time is too long (exceeding 5 hours), the fibers may become over-oxidized, leading to increased brittleness and a marked reduction in tensile strength.

#### 4.2.3 Influence of atmospheric environment

During stabilization, the oxygen concentration is typically regulated within the range of 5–21%. Higher oxygen levels can accelerate the oxidation rate but must be managed carefully to avoid excessive exothermic reactions that may damage fiber morphology.

In addition, the gas flow rate must be optimized to ensure uniform oxygen diffusion throughout the fiber tow. Uneven oxygen distribution can lead to localized oxygen depletion or overheating, which compromises oxidation uniformity. A well-designed gas circulation system with no dead zones is essential to maintain atmospheric homogeneity, which in turn improves the continuity and consistency of the final carbon fibers.

### 4.3 Influence of environmental and operational factors on stabilization treatment

In addition to material properties and processing parameters, environmental and operational factors play a critical role in determining the uniformity and effectiveness of the pre-oxidation (stabilization) process. The main considerations are as follows:^117,118^

#### 4.3.1 Sample morphology and packing density

During pre-oxidation, fiber morphology and placement significantly affect oxidation efficiency. Filamentous or planar fibers should be loosely arranged to prevent adhesion between individual filaments. Bulk materials, on the other hand, should be processed in thin layers to allow adequate gas exposure.

It is also essential to limit fiber packing density, as excessive compaction can impede oxygen diffusion, particularly into the inner regions of the fiber bundles. This may result in incomplete internal oxidation, leading to structural inconsistencies and degraded carbon fiber quality.

#### 4.3.2 Oxidation equipment design

Pre-oxidation is commonly performed using either a hot-air circulation reactor or a static furnace. Among these, hot-air circulation systems provide superior uniformity of temperature and gas distribution, which is critical for consistent crosslinking across the entire fiber volume.

During operation, the direction of gas flow should be aligned as closely as possible parallel to the fiber orientation to enhance contact efficiency and promote uniform oxidation throughout the fiber length.

#### 4.3.3 Pre oxidation degree monitoring

The termination indicators of the pre-oxidation reaction include: a fiber weight gain rate reaching the target range of 5–10%, an increase in softening point to above 300 °C, and no melting of the fibers. The end-point control can be achieved by monitoring the intensity of the C=O carbonyl peak using FT-IR, or by observing changes in the exothermic peak using DSC; if necessary, the data can be fitted and analyzed to support judgment.

### 4.4 Reaction mechanism-related factors

The pre-oxidation process is influenced by several key reaction mechanisms, which are outlined as follows:^119–121^

#### 4.4.1 Selectivity of crosslinking reactions

The core objective of pre-oxidation is to promote dehydrogenation crosslinking reactions between fiber molecules, while simultaneously suppressing molecular cleavage reactions to avoid the volatilization of low-molecular-weight by-products. The weight loss of the fiber structure should be controlled within 5%. To prevent excessive crosslinking, inhibitors such as B_2_O_3_ can be added to moderate free radical activity and stabilize the reaction.

#### 4.4.2 Oxygen diffusion kinetics

In the pre-oxidation process, the oxygen diffusion reaction in the fibers follows Fick's law of diffusion, where the oxidation layer thickness 
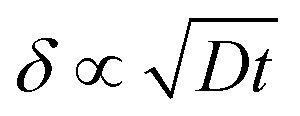
 (*D*: diffusion coefficient). As the temperature increases, *D* increases, but the crosslinking rate also accelerates, which may require optimization of other parameters to maintain balance.

### 4.5 Common issues and mitigation strategies

Common problems encountered during the pre-oxidation process, along with their root causes and proposed solutions, are as shown in Table S2:^122–124^

### 4.6 Optimization strategies

To address the challenges discussed in Sections 4.1–4.5, the following optimization approaches may be considered:^125–128^

#### 4.6.1 Stepwise oxidation process

(1) Low-temperature stage (200–230 °C): promotes slow formation of a stable crosslinked network.

(2) Medium-temperature stage (240–280 °C): acts as the main reaction zone; oxygen concentration is regulated to accelerate crosslinking.

(3) High-temperature stage (280–300 °C): a short holding period is applied to complete deep structural curing.

#### 4.6.2 Additive-based modification

(1) Introduction of phosphate esters can lower the oxidation activation energy, reducing processing time by more than 30%.

(2) Incorporation of nano-silica (SiO_2_) enhances thermal conductivity, helping to minimize temperature gradients between the inner and outer fiber regions.

#### 4.6.3 Online monitoring technology

Real-time analytical tools such as TGA and FT-IR can be used to monitor volatile components, allowing for precise determination of the reaction endpoint and improved process control.

### 4.7 Chapter summary

The content and morphology of mesophase pitch play a decisive role in shaping both the uniformity and efficiency of the oxidation reaction, thereby directly influencing the design strategy of the stabilization process and the performance of the resulting carbon fibers. In practical production, the preparation of high-performance fibers with controllable structures can be achieved through the coordinated optimization of raw material selection, morphological control (such as spinning process refinement), and stabilization parameters including temperature, duration, and atmospheric conditions.

The effectiveness of the stabilization process largely depends on the synergistic interaction among raw materials, processing techniques, and equipment. From the perspective of materials, pitch with low quinoline-insoluble (QI) content and a narrow molecular weight distribution is preferred. In terms of process design, strategies such as gradient temperature ramping, stepwise oxygen concentration control, and precise timing are critical. As for equipment, the use of a forced-convection hot-air furnace is essential to ensure uniform heat and gas distribution throughout the fiber assembly.

By finely regulating these factors, it is possible to achieve high stabilization yield with minimal structural defects, thereby producing an ideal precursor for subsequent carbonization and enabling the development of high-performance carbon fibers.

## 5. Introduction to carbonization treatment

After stabilization, the precursor fibers must undergo carbonization under an inert nitrogen atmosphere. The primary purpose of this stage is to promote further crosslinking, polycondensation, and aromatization within the internal structure of the fibers, while gradually removing non-carbon elements in the form of gaseous by-products. This leads to the formation of aromatic planar networks with a graphite-like structure.^129–131^

**Fig. 5:**
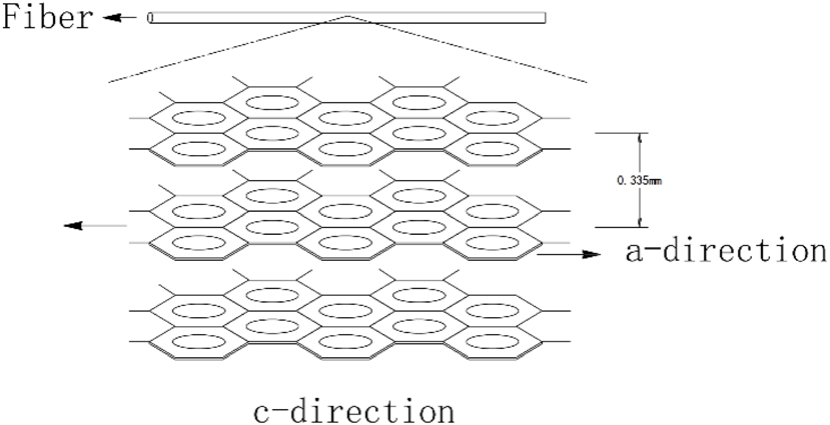
Ideal carbon fiber structure.

As the temperature continues to rise, the internal structure of the fibers progressively transforms into an ordered graphite-like arrangement. The interlayer spacing decreases, and crystallite size increases. Once the temperature exceeds a certain critical threshold, the oxygen content in the fibers drops significantly, resulting in the formation of the primary carbon fiber structure, as illustrated in Fig. 5.^132^ This phase is dominated by dehydrogenative condensation reactions, primarily occurring between aromatic ring structures to form large planar aromatic molecules. With continued heating, these aromatic planes grow in size, eventually forming a turbostratic graphite-like structure around 1000 °C, characterized mainly by sp^2^-hybridized carbon atoms.

The carbonization of mesophase pitch is a critical transformation stage, where the stabilized mesophase pitch is converted into carbon material. The effectiveness of this process directly influences the degree of graphitization, mechanical properties, electrical conductivity, and microstructure of the final carbon fibers. The following sections analyze the key factors and mechanistic influences affecting the carbonization process.

In the production of mesophase pitch-based carbon fibers, carbonization is a key thermal treatment step, in which the stabilized fibers are heated to 1000–1600 °C under an inert atmosphere. The main objective is to eliminate non-carbon elements such as hydrogen, oxygen, and nitrogen, and to form the initial disordered graphite structure, which serves as the structural foundation for subsequent graphitization.^133–135^

The extent and quality of carbonization directly affect the final fiber's strength, density, porosity, and graphitization potential. The following section presents the critical factors that influence carbonization performance, along with strategies for process optimization.

### 5.1 Key factors influencing the effectiveness of carbonization treatment

#### 5.1.1 Properties of fibers after stabilization

The quality of carbonization is significantly influenced by the performance of the fibers following stabilization. The key evaluation indicators and influencing factors are as follows:^136–139^

##### 5.1.1.1 Degree and uniformity of crosslinking

The oxygen-bridge crosslinked network formed during stabilization must be uniform and comprehensive. Insufficient crosslinking can lead to problems such as fiber fusion, deformation, or even breakage during carbonization. Conversely, excessive or uneven crosslinking may cause localized stress concentrations, resulting in cracks and internal defects within the fiber structure.

##### 5.1.1.2 Retention of molecular orientation

The molecular orientation established during melt spinning should be preserved as much as possible throughout the stabilization stage. A high degree of retained orientation facilitates the formation of more ordered microcrystalline structures during carbonization, thereby promoting more effective subsequent graphitization.

##### 5.1.1.3 Oxygen content and distribution

Oxygen is a key element introduced during stabilization. Its content and distribution uniformity directly affect the intensity and homogeneity of deoxygenation and dehydrogenation reactions during carbonization. Excessive localized oxygen content in the fiber structure can lead to violent exothermic reactions, increasing the risk of structural defects such as pore formation or microcracking.

#### 5.1.2 Carbonization process parameters

The key parameters of the carbonization process are described as follows:^140–144^

##### 5.1.2.1 Temperature program (heating rate, final temperature, and holding time)

(1) Low-temperature stage (over the temperature range of 25–600 °C):

During this phase, pyrolysis reactions dominate, including dehydrogenation, deoxygenation, and the removal of volatile small molecules such as tar, H_2_, CH_4_, CO, and CO_2_. The heating rate is the critical control factor. If the heating rate is too rapid, it may cause violent volatile release, leading to internal pressure buildup, which can result in porosity, cracking, or even fiber blistering. It is also essential to avoid excessive internal–external temperature gradients, which may lead to thermal stress-induced fiber fracture. Ensuring a uniform thermal response is crucial for minimizing structural defects.

**Fig. 6:**
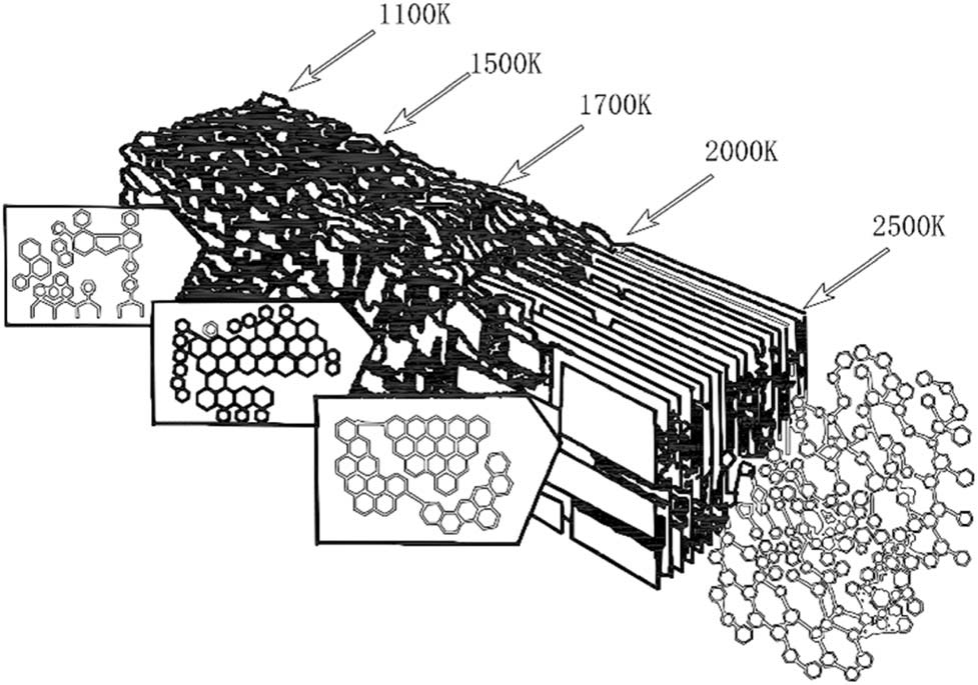
The trend of changes in the internal carbon layer structure of carbon fibers during heat treatment.

(2) Intermediate-temperature stage (600–1000 °C): in this range, aromatization and polycondensation reactions occur. Basic Structural Units (BSUs) of turbostratic carbon begin to form and grow. The heating rate must be carefully controlled to prevent excessive release of residual volatiles, which could otherwise damage the developing fiber structure.

(3) High-temperature stage (ranging from above 1000 °C up to the designated terminal temperature):

At this stage, graphitic microcrystalline structures continue to grow, and the carbon structure becomes increasingly dense. Generally, higher final temperatures promote the formation of larger crystallites, higher material density, and greater carbon content. However, a balance must be maintained between performance optimization and energy consumption. The duration of the final temperature holding period significantly influences the extent of structural evolution. Fig. 6 illustrates the evolution of internal carbon layer structures in carbon fibers during thermal treatment.^144^

##### 5.1.2.2 Atmosphere and flow rate

Inert gases such as high-purity nitrogen (N_2_) or argon (Ar) are typically used as the protective atmosphere during carbonization. It is critical to completely isolate the system from oxygen to prevent oxidative degradation. The impurity levels must be strictly controlled, especially O_2_ below 10 ppm and H_2_O below 5 ppm. A sufficiently high gas flow rate is essential for promptly removing pyrolysis volatiles, particularly tar. This helps to avoid secondary cracking and deposition of volatiles on fiber surfaces, which could lead to the formation of a pyrolytic carbon layer, degrading fiber performance or even clogging the furnace tube. However, an excessively high flow rate will increase energy consumption and operational costs.

##### 5.1.2.3 Tension control

Applying tensile force during carbonization serves to prevent fiber shrinkage at high temperatures and to maintain or enhance molecular orientation, thereby suppressing fiber deformation or bending. Nevertheless, care must be taken during the low-temperature stage (<600 °C) when fibers exhibit low mechanical strength due to rapid weight loss and structural transformation. Excessive tension at this stage can easily cause fiber breakage, so the tension must be dynamically adjusted in response to temperature changes throughout the process.

#### 5.1.3 Equipment factors

The experimental equipment factors that influence the carbonization process are as follows:^145,146^

##### 5.1.3.1 Furnace type and temperature zone design

Multi-zone tubular furnace is recommended, allowing independent temperature control in each zone and enabling a precise thermal gradient. The length and number of zones should be designed to match the peak volatilization temperature ranges. Materials for furnace tubes should be selected for high-temperature resistance, such as Inconel alloys or quartz. The tube diameter must ensure uniform gas flow to avoid “chimney effects” that can cause uneven temperature or atmosphere distribution.

##### 5.1.3.2 Temperature uniformity

Uniformity of temperature in both the radial and axial directions of the furnace is critical. Inconsistent temperatures may lead to variation in fiber properties within fiber bundles or between different furnace positions.

##### 5.1.3.3 Off-gas exhaust system

An efficient exhaust system is essential, including side extraction ports and a well-designed negative pressure system, to ensure the rapid removal of volatiles and prevent tar condensation. In addition, the system should be equipped with tar traps and waste gas treatment units to handle emissions effectively.

### 5.2 Systematic optimization strategies for carbonization treatment

#### 5.2.1 Ultimate optimization of precursors and stabilization

The carbonization process imposes strict requirements on the quality of precursors. The following aspects are crucial for optimization:^147,148^

##### 5.2.1.1 Stabilization process

It is essential to optimize the pre-oxidation conditions, including temperature, duration, oxygen concentration in the atmosphere, and humidity, to ensure that molecular cross-linking within the fiber is both sufficient and uniform. A programmed temperature-ramping oxidation strategy should be adopted, tailored to the softening point and chemical reactivity of the pitch. The use of mild oxidants—such as NO_2_, ozone, or plasma treatment—may further improve the homogeneity of molecular cross-linking.

##### 5.2.1.2 Fiber quality monitoring

The stabilized fibers must undergo rigorous quality control to monitor parameters such as oxygen content distribution, density, mechanical properties, and thermal behavior. These measures are necessary to ensure that fiber performance aligns with experimental records and target specifications.

#### 5.2.2 Fine control of carbonization process parameters

The precision control of carbonization parameters involves the following key operations:^149–151^

##### 5.2.2.1 Customized heating program

(1) In the low-temperature stage (typically ranging from room temperature to approximately 600 °C), which is critical to the carbonization process, a slow heating rate is generally applied, usually between 0.5–2 °C min^−1^. Stepwise isothermal holds are often introduced, especially at the peak temperatures of volatile release A typical protocol includes heating at 1 °C min^−1^ from room temperature to 350 °C, followed by a 30–60-minute isothermal hold, then continuing to 450 °C at the same rate, again followed by a 30–60-minute hold. This approach ensures a smooth and complete release of volatiles.

(2) In the mid- to high-temperature stage (above 600 °C), the heating rate may be increased moderately to 5–10 °C min^−1^, while still avoiding excessively rapid heating that could compromise structural integrity.

##### 5.2.2.2 Final temperature and holding time

The final carbonization temperature is selected based on the targeted grade of the carbon fiber product, generally ranging between 1000–1600 °C. A holding time of 10–30 minutes at the final temperature is typically applied to stabilize the fiber structure. Higher terminal temperatures are preferred for achieving high-density, high-strength carbon fibers.

##### 5.2.2.3 Atmosphere system optimization

The purity of the gaseous environment is critical. Ultra-high purity inert gases such as N_2_ or Ar should be used, with oxygen and water vapor contents maintained below 1 ppm. Online gas purifiers are recommended. The gas flow direction should be optimized using a countercurrent design, where fresh gas enters from the low-temperature zone and exits at the high-temperature zone. This ensures the cleanest gas comes into contact with the hottest fibers, maximizing protective effects. Flow rates must be calculated and optimized based on reactor tube size, fiber loading, and volatile production, with linear velocities typically exceeding 0.1 m s^−1^. Exhaust composition should be continuously monitored.

##### 5.2.2.4 Intelligent tension control

In the low-temperature region (room temperature to ∼600 °C), extremely low or zero tension should be applied to keep the fibers straight and prevent breakage, preferably with low-friction guide rollers. As the temperature increases (generally >800 °C) and the fibers regain strength, moderate tension can be gradually introduced. This helps suppress shrinkage, improve molecular orientation, and can be managed *via* a closed-loop tension control system that dynamically adjusts based on real-time feedback on fiber shrinkage.

#### 5.2.3 Equipment upgrades and process monitoring

In response to the challenges encountered in the carbonization process, the following targeted optimization strategies are proposed:^152,153^

##### 5.2.3.1 Furnace design

The number and length of temperature zones in the low-temperature section can be appropriately increased to ensure sufficient volatilization of light components and adequate isothermal holding time. It is essential to optimize thermal field uniformity by employing high-precision temperature controllers, improving the layout of heating elements, enhancing thermal insulation layers, and regularly calibrating furnace temperature uniformity, keeping deviations within ±10 °C. For sections prone to tar accumulation, furnace tubes with internal linings or special coatings may be used. Alternatively, self-cleaning structures can be designed to suit the equipment characteristics.

##### 5.2.3.2 Efficient exhaust gas treatment

A combination of multistage condensation, electrostatic precipitation, incineration, and catalytic oxidation is recommended to thoroughly remove tars and hazardous gases. This not only ensures environmental compliance but also prevents clogging and contamination of furnace tubes.

##### 5.2.3.3 Real-time online monitoring

Integrated fiber-optic thermometry can be employed to monitor the fiber temperature in real time. Gas analyzers may be used to track the release patterns of CO, CO_2_, CH_4_, and other volatile components. Additionally, sensors for tension and diameter measurement can enable closed-loop control of the entire process, ensuring stability and quality during carbonization.

#### 5.2.4 Exploration of innovative technologies

Beyond the technological optimizations detailed in Section 5.2.3, emerging carbonization methods are being actively explored. Promising directions include:^154–157^

(1) Pressurized carbonization, conducted under a moderate positive-pressure inert atmosphere, which can suppress pore formation and promote fiber densification. Although this technique remains under refinement, it shows great potential.

(2) Microwave-assisted carbonization, where the fiber bundle is uniformly heated from within, reducing internal temperature gradients and thermal stress while shortening processing time. However, issues such as uneven microwave absorption across fiber bundles must be resolved.

(3) Catalytic carbonization, which introduces trace amounts of transition metal catalysts to lower the activation energy for aromatization and polycondensation reactions, thus facilitating the formation of ordered structures at lower temperatures. This approach requires strict control over catalyst type and residual levels to prevent contamination.

### 5.3 Chapter summary

Synthesizing insights from Sections 5.1 and 5.2, it is evident that the key to optimizing the carbonization of mesophase pitch-based carbon fibers lies in controlling the intensity and uniformity of pyrolytic reactions. This helps minimize structural defects such as pores, cracks, and loss of molecular orientation, ultimately leading to the formation of dense, highly oriented turbostratic carbon structures. Major conclusions can be summarized as follows:

(1) Temperature ramping control: in the low-temperature zone where volatile components are released, a slow heating rate combined with appropriate isothermal treatments effectively reduces structural defects.

(2) Purity of reaction environment: the use of ultra-high-purity gases, high flow rates, optimized flow direction, and efficient tar removal ensures a clean environment that prevents secondary contamination.

(3) Stability of the precursor: a stable pre-oxidized fiber is a prerequisite for successful carbonization. Intelligent tension strategies that adjust dynamically with temperature zones can maintain fiber orientation and morphology.

(4) Uniformity of experimental conditions: maintaining high uniformity in the thermal, atmospheric, and stress fields helps ensure consistent fiber bundle properties.

At lower heating rates (1–2 °C min^−1^), molecular rearrangement is more ordered and internal stress is reduced, significantly improving fiber strength. In contrast, higher heating rates (>5 °C min^−1^) lead to rapid volatile release, causing closed-pore structural defects. While high-purity nitrogen (N_2_) is cost-effective and commonly used as a protective gas, it may generate cyanides and corrode equipment at temperatures above 1800 °C. For carbonization temperatures exceeding 1500 °C, argon (Ar) with a purity >99.999% is recommended due to its superior inertness and ability to suppress high-temperature gasification reactions.

When fibers are in the intermediate temperature range (800–1200 °C), applying a stretch ratio of 5–10% helps align carbon layers axially, increasing the elastic modulus by more than 15%. In the high-temperature zone (>1800 °C), a relaxation ratio of 0–2% is recommended to relieve thermal stress and prevent fiber breakage.

Through the integration of refined process control, equipment upgrades, and intelligent monitoring, significant improvements can be made in carbon fiber density, strength, structural uniformity, and graphitization potential. These enhancements lay a solid foundation for the production of high-performance carbon fibers. Continuous process data analysis and feedback serve as the core driving force for ongoing optimization.

## 6. Graphitization treatment overview

Mesophase pitch-based carbon fibers exhibit excellent molecular orientation, largely attributed to the graphitization process.^158–160^ When the thermal treatment temperature exceeds 1800 °C, the degree of order in the graphite microcrystals within the fiber structure increases significantly. At temperatures above 2000 °C, structural defects and misalignments within the lamellar planes nearly vanish, and the graphite microstructure gradually transforms into a three-dimensional ordered graphitic arrangement. This structural evolution leads to enhanced thermal and electrical conductivity, increased density, and a marked improvement in both tensile modulus and strength.

**Fig. 7:**
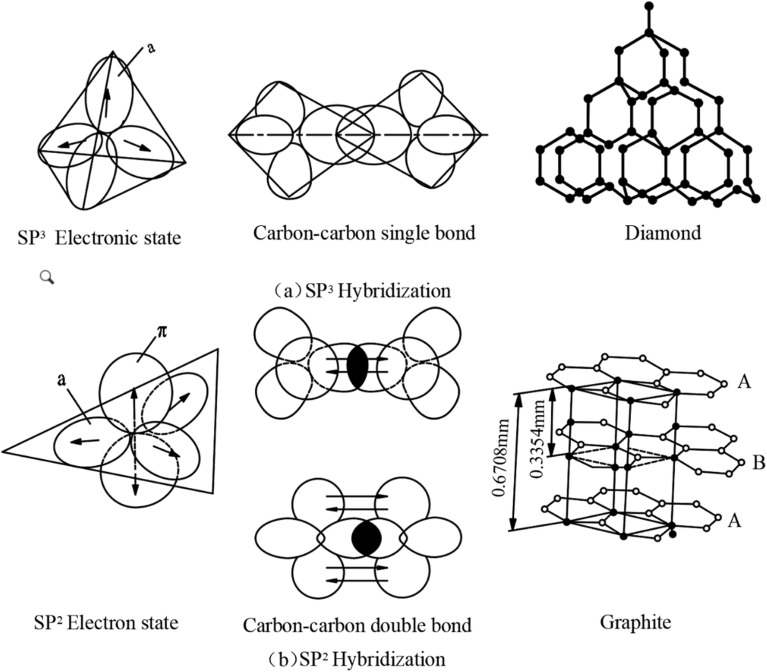
Schematic diagram of molecular structure of diamond and graphite.

In the fabrication of mesophase pitch-based carbon fibers, graphitization is a critical step for enhancing fiber modulus, thermal conductivity, and electrical conductivity. The process typically involves heat treatment above 2500 °C, enabling the rearrangement of disordered graphite structures into highly ordered three-dimensional graphite crystals. A schematic comparison between diamond and graphite molecular structures is shown in Fig. 7.^132,161^

The effectiveness of the graphitization process is influenced by numerous factors, including the properties of the precursor material, process parameters, and equipment conditions—all of which must be comprehensively optimized to achieve high-performance fibers.

### 6.1 Key factors affecting graphitization efficiency

#### 6.1.1 Quality of precursor fibers

The factors that affect the quality of precursor fibers are as follows:^162–166^

##### 6.1.1.1 Degree of molecular orientation

Molecular orientation achieved during spinning and drawing serves as the structural foundation for graphitization. The higher the orientation, the more readily microcrystals grow and align along the fiber axis during graphitization, directly contributing to the enhancement of fiber modulus.

##### 6.1.1.2 Content and purity of mesophase

Pitch precursors with high mesophase content and purity contain a greater proportion of easily graphitizable polycyclic aromatic structures, which enhances the potential for effective graphitization. In contrast, solid impurities such as quinoline-insoluble matter and ash hinder graphitic transformation and introduce structural defects.

##### 6.1.1.3 Structure of carbonized fibers

**Fig. 8:**
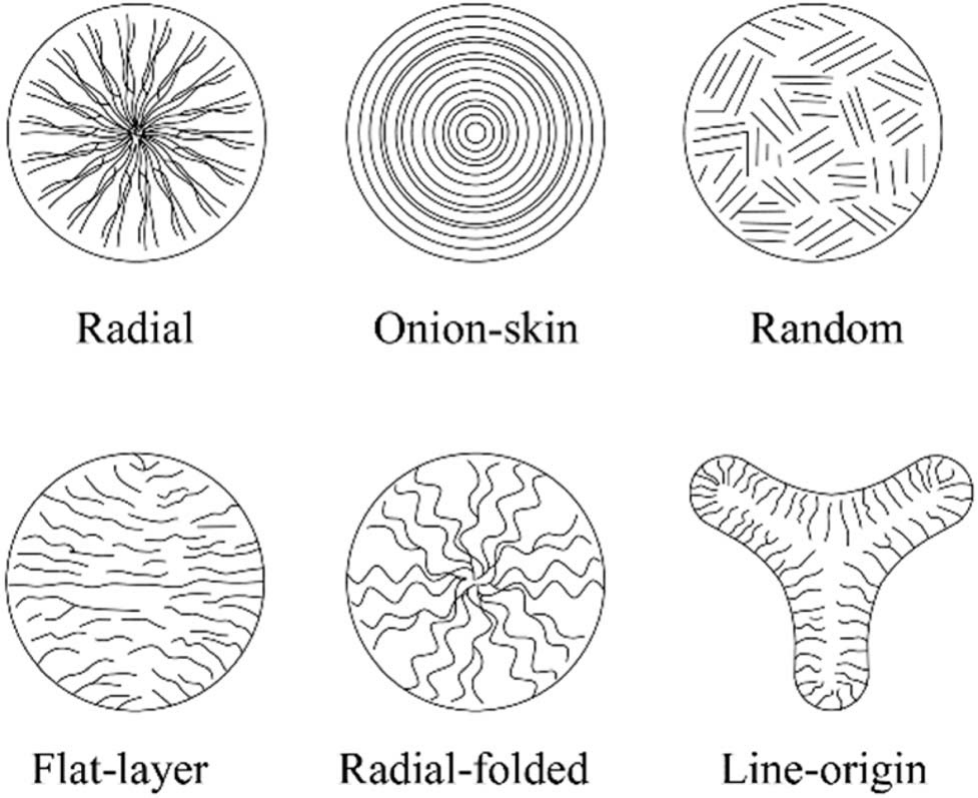
Cross sectional structural morphology of several common mesophase pitch-based carbon fibers.

**Fig. 9:**
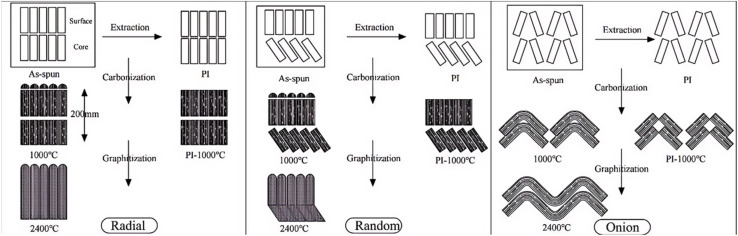
Preparation process of radial, disordered, and onion shaped carbon fibers.

The porosity, microcrystal size, and degree of disorder in the carbonized fiber directly affect the onset and efficiency of graphitization. Carbon fibers with high density and large microcrystalline domains are more conducive to the development of graphitic order.^165^Fig. 8 illustrates the cross-sectional morphologies of several typical mesophase pitch-based carbon fibers. Among them, fibers with radial, disordered, and onion-like structures exhibit superior integrated properties. The specific fabrication processes for these structures are detailed in Fig. 9.^166^

#### 6.1.2 Graphitization process parameters

The effectiveness of the graphitization process is significantly influenced by key process parameters, as outlined below:^167–169^

##### 6.1.2.1 Maximum heat treatment temperature

The peak temperature during thermal treatment—typically the final process temperature—is the most critical parameter. Higher final temperatures enhance carbon atom mobility, which facilitates defect elimination, increases microcrystal size (especially the stacking height along the *c*-axis, *L*_c_), and improves the degree of graphitization. To achieve high-modulus fibers, temperatures of 2800 °C or even above 3000 °C are generally required. Insufficient temperatures may result in incomplete graphitization.

##### 6.1.2.2 Soaking time at maximum temperature

Maintaining the target temperature for an adequate duration allows for structural rearrangement and microcrystal growth to reach equilibrium. Insufficient soaking time results in incomplete structural optimization, whereas excessive soaking can lead to abnormal grain growth or reduced mechanical strength due to defect propagation or volatile loss. Additionally, prolonged soaking increases energy consumption. The optimal duration depends on the final temperature, fiber properties, and furnace atmosphere.

##### 6.1.2.3 Heating rate

During the low-temperature stage (<1500 °C), a slow heating rate (typically 5–10 °C min^−1^) is recommended to ensure the gradual release of residual volatiles and uniform stress relief, thus minimizing the formation of cracks and pores. If the heating rate is too fast, volatile compounds are rapidly expelled, causing internal pressure buildup and defects such as bubbling, cracking, or deformation. Moreover, these volatiles may decompose in the furnace and form carbon deposits, which can contaminate fiber surfaces or clog the system.

In the high-temperature stage (>2000 °C), a faster heating rate (>20 °C min^−1^) can improve efficiency, but may result in significant temperature gradients between the fiber's interior and exterior, generating thermal stress and potentially damaging the fiber structure. A balance must be found between processing efficiency and structural integrity.

##### 6.1.2.4 Cooling rate

An excessively rapid cooling rate may “freeze” unstable structures formed at high temperatures or induce thermal stress, leading to microcracks within the fiber. Controlled cooling is typically employed to avoid such issues.

##### 6.1.2.5 Reaction atmosphere

Graphitization must be carried out in an inert atmosphere, with high-purity argon (Ar) being the most commonly used. Oxygen must be strictly excluded to prevent oxidative degradation of the fibers. Nitrogen is unsuitable, as it reacts with carbon at temperatures above 2000 °C to form cyanide species, which severely damage fiber structure and corrode equipment. The purity of the atmosphere is crucial—trace levels of impurities (especially O_2_, H_2_O, and N_2_) must be strictly controlled, as even minimal contamination can inhibit graphitization or introduce structural defects. A high gas flow rate is beneficial to remove volatile impurities and reaction by-products efficiently.

#### 6.1.3 Physical state and stress of fibers

Recent research has indicated that the physical state and applied stress on carbon fibers can significantly influence their mechanical properties. The main influencing factors and underlying mechanisms are described below:^170,171^

##### 6.1.3.1 Application of tension

Applying appropriate axial tension during graphitization is a key strategy for enhancing the degree of graphitization and the modulus of the fibers. This tension helps align the graphene layers along the fiber axis, increases molecular orientation, suppresses thermal shrinkage or deformation at high temperatures, and assists in eliminating structural defects. However, the magnitude of tension must be precisely controlled: if too low, the effect is negligible; if too high, it may exceed the fiber's tensile strength at elevated temperatures and result in fiber breakage. Tension should be dynamically adjusted in accordance with the temperature rise during processing.

##### 6.1.3.2 Fiber relaxation or shrinkage

If no tension or insufficient tension is applied, the fiber tends to undergo thermal shrinkage at high temperatures, leading to a decrease in molecular orientation. This negatively impacts the development of high-modulus fibers.

#### 6.1.4 Equipment factors

Experimental equipment also plays a crucial role in the graphitization process. Key equipment-related factors include:^172,173^

##### 6.1.4.1 Furnace type and heating elements

The most commonly used furnace is the graphite resistance furnace, often combined with graphite tubes, rods, and felt. This setup is relatively cost-effective and easy to maintain. However, at high temperatures (>2500 °C), graphite components may sublimate and release carbon vapor, which can deposit on fiber surfaces, forming a pyrolytic carbon layer or contaminating the furnace. This necessitates extremely high atmosphere purity. Alternatively, induction furnaces offer rapid heating, better temperature uniformity, and no electrode degradation issues, though they are more complex and expensive, and still face volatilization concerns. Advanced technologies such as plasma furnaces or laser heating systems, which allow for ultra-high temperatures within short durations, offer promising results but are still under development due to high cost and technical complexity.

##### 6.1.4.2 Temperature uniformity

Uniform temperature distribution within the effective high-temperature zone of resistance furnaces is essential for consistent fiber performance. Uneven heating can result in significant variations in the degree of graphitization across the fiber bundle, hindering the production of structurally uniform graphite fibers.

##### 6.1.4.3 Furnace cleanliness

Maintaining a clean furnace environment is critical. Residual carbon deposits or impurities inside the chamber may contaminate the fibers or compromise the purity of the reaction atmosphere.

### 6.2 Optimization strategies for graphitization treatment

#### 6.2.1 Optimization of precursors and pretreatment

High-quality pitch precursors with a high mesophase content and low impurity levels (particularly low quinoline insolubles and low ash content) should be selected.^174^ The spinning, drawing, and stabilization processes must be optimized to maximize molecular orientation and fiber densification. Furthermore, refining the carbonization process is essential to produce carbon fibers with uniform structure, appropriate microcrystal size, and minimal defects. In the later stages of carbonization, introducing a slightly elevated temperature “pre-graphitization” treatment can be considered to enhance structural evolution.

#### 6.2.2 Precise control of graphitization parameters

The following graphitization process parameters require strict and accurate control:^175,176^

##### 6.2.2.1 Graphitization reaction temperature

The final temperature should be determined based on the target modulus grade, typically ranging from 2800 to 3000 °C. Accurate temperature measurement is critical, and the use of optical pyrometers or tungsten-rhenium thermocouples is recommended.

##### 6.2.2.2 Graphitization holding time

The optimal dwell time at the target temperature should be determined experimentally to balance performance improvement with energy consumption and processing efficiency.

##### 6.2.2.3 Heating profile

A multi-stage heating schedule is recommended: in the low-temperature range (<1500 °C), a slow heating rate (*e.g.*, 5–10 °C min^−1^) allows for thorough degassing and stress release. In the mid-temperature range (1500–2500 °C), the rate may be increased appropriately (*e.g.*, 20–50 °C min^−1^), then slowed again as the target temperature is approached. Where feasible, advanced rapid-heating techniques such as induction heating or plasma-assisted heating can be employed in the high-temperature range to improve efficiency and potentially achieve unique structural characteristics.

##### 6.2.2.4 Cooling process

A controlled, programmed cooling process is recommended, particularly in critical temperature ranges (typically above 2000 °C), to regulate the cooling rate and avoid internal stress or structural degradation.

##### 6.2.2.5 Reaction atmosphere

Ultra-high-purity inert gases (*e.g.*, 6N-grade argon) should be used to maintain a stable and sufficiently high flow rate. The atmosphere must be regularly monitored and purified. In specific stages, trace amounts of dopant gases (*e.g.*, B_2_H_6_) may be introduced to facilitate catalytic graphitization.

#### 6.2.3 Optimization of tension control

Recent studies have shown that tension control during the graphitization process plays a critical role in the production of high-performance carbon fibers.^177^

##### 6.2.3.1 Application of tension

Applying axial tension during graphitization is a key step in achieving high-modulus pitch-based carbon fibers. This process helps align graphene layers along the fiber axis, enhancing molecular orientation and promoting a more ordered graphite structure.

##### 6.2.3.2 Control of tension magnitude

Optimal tension values should be determined experimentally for different temperature zones. Typically, lower tension is applied in the early stages of graphitization to avoid fiber breakage. The maximum tension should be applied at the highest temperature stage, ideally approaching but not exceeding the tensile strength of the fiber at that specific temperature.

##### 6.2.3.3 Tension application modes

Different tension control strategies—such as constant tension and variable tension—should be investigated to assess their effects. Additionally, the development of precise online tension monitoring and feedback control systems is recommended to maintain consistent and optimal tension throughout the process.

##### 6.2.3.4 Method of tension application

It is essential to ensure that the tension is applied uniformly across the entire fiber bundle to avoid uneven stress distribution among individual filaments, which can compromise fiber quality.

#### 6.2.4 Equipment upgrades and maintenance

The following strategies can be considered to improve equipment performance and enhance graphitization efficiency:^178–180^

##### 6.2.4.1 Selection of furnace type

For the targeted production of ultra-high modulus (typically ≥900 GPa) and high thermal conductivity carbon fibers, advanced furnace types such as induction furnaces may be considered. These systems help reduce contamination caused by graphite vaporization at extreme temperatures.

##### 6.2.4.2 Heating elements and insulation materials

High-purity, high-density, and low-volatility graphite materials—such as isostatic graphite—are recommended for use as heating elements and thermal insulation. Regular inspection and timely replacement of these materials are essential to maintain furnace performance.

##### 6.2.4.3 Temperature uniformity

Optimizing furnace chamber design, including adopting muffle structures, strategic arrangement of heating elements, multi-zone temperature control, and precision temperature measurement techniques, can significantly improve temperature uniformity. It is advisable to maintain the temperature deviation within the effective high-temperature zone to within ±20 °C of the target temperature.

##### 6.2.4.4 Atmosphere system

A high-efficiency gas purification system should be integrated, including oxygen, moisture, and particulate filters, to ensure ultra-high-purity inert gas input. Additionally, optimizing the gas flow field within the furnace is crucial to achieve full gas renewal and eliminate dead zones.

##### 6.2.4.5 Furnace cleaning

Regular cleaning of the furnace is necessary. This may involve high-temperature burn-off under an inert atmosphere to remove deposits or thorough mechanical cleaning. Maintaining a clean furnace chamber is essential for ensuring consistent fiber quality and preventing contamination.

#### 6.2.5 Introduction or development of new technologies as needed

To achieve better experimental outcomes, it is worth considering the adoption or development of novel technologies. Several promising directions are outlined below:^181–183^

(1) Catalytic graphitization: research has explored introducing small amounts of catalysts—such as boron, titanium, iron, or their compounds—into the fiber structure or reaction atmosphere. These catalysts aim to lower the activation energy of graphitization, enabling higher degrees of graphitization at relatively lower temperatures or within shorter durations. However, the type, concentration, and method of catalyst introduction must be precisely controlled to avoid unwanted side reactions.

(2) High-pressure graphitization: performing graphitization under high-pressure inert gas conditions may help suppress graphite sublimation and promote structural ordering. However, this approach involves complex and costly equipment and remains under active development.

(3) Alternative heating methods: techniques such as microwave heating or plasma-assisted heating may offer more efficient and uniform thermal environments, representing future directions for process innovation.

### 6.3 The important applications of carbon fiber

The main application areas of carbon fiber can be roughly summarized as shown in Table S3.^175–177^

#### 6.3.1 Core summary

The applications of mesophase pitch-based carbon fibers are almost exclusively concentrated in high-end, high value-added fields that demand extreme performance in thermal/electrical conductivity, dimensional stability, and rigidity. This distinguishes them from PAN-based carbon fibers, which are primarily optimized for high strength and toughness, thereby highlighting a complementary differentiation in functional applications.

### 6.4 Chapter summary

Optimizing the graphitization process for mesophase pitch-based carbon fibers is a critical step in enhancing their performance. The core principles include:

① Providing sufficiently high temperatures and appropriate reaction durations.

② Applying precisely controlled axial tension in high-temperature zones to maximize molecular orientation and tensile modulus.

③ Maintaining a highly pure inert gas atmosphere throughout the process.

④ Using high-quality precursor fibers with high molecular orientation and minimal structural defects—primarily derived from well-refined pitch.

Optimization strategies should revolve around these principles, involving the meticulous control of process parameters—such as temperature profiles, tension schemes, and atmospheric conditions—alongside equipment upgrades. It is also crucial to ensure system uniformity, maintain a clean processing environment, and explore auxiliary measures like catalytic additives. All these efforts should aim to maximize graphitization degree, crystallite size, and orientation without compromising fiber integrity or production efficiency. Effective process monitoring, data analysis, and experimental validation are essential for achieving optimal outcomes.

## 7. Discussion

Compared with laboratory-scale studies, industrial-scale melt spinning faces three core challenges:^184–187^

(1) Maintaining a uniform temperature field is essential, since fluctuations beyond 5 °C can result in measurable deviations in fiber fineness.

(2) In multi-hole spinnerets, shear rate gradients are observed to induce flow velocity differences of 15–20% when the nozzle count exceeds 1000 at an industrial scale.

(3) Localized overheating caused by heat accumulation during the oxidation process, which necessitates the development of graded temperature control systems.

## 8. Future outlook

According to previous literature, current research efforts can be initiated from the following aspects:^184–191^ green raw materials, intelligent manufacturing, and precision-controlled microstructures. Through the comprehensive optimization of mesophase pitch synthesis, melt spinning, stabilization, carbonization, and graphitization processes, the production of mesophase pitch-based carbon fibers can simultaneously improve experimental efficiency, reduce costs, enhance safety, and boost final product performance.

## Conflicts of interest

Authors declare no competing interest.

## Supplementary Material

RA-015-D5RA05325K-s001

## Data Availability

No primary research results, software or code have been included and no new data were generated or analysed as part of this review. Supplementary information: Table S1. The market share of carbon fiber within the current time frame. Table S2. Common issues and solutions in the pre-oxidation process of carbon fibers. Table S3. Summary of key application fields of mesophase pitch-based carbon fibers. See DOI: https://doi.org/10.1039/d5ra05325k.
