# Supplementary material for: Preparation process of mesophase pitch-based carbon fiber: a review
Source: RSC Adv. 2025 Oct 20;15(47):39514–38. doi: 10.1039/d5ra05325k (PMC12536871; doi:10.1039/d5ra05325k)
Supplement: RA-015-D5RA05325K-s001 [file RA-015-D5RA05325K-s001.pdf]

Supplementary Information (SI) for RSC Advances.
This journal is © The Royal Society of Chemistry 2025

Table.S1. The market share of carbon fiber within the current time frame.

Manufacturer	Trade Name	Production Capacity (t/a)	Pitch Raw Material	Product Type	Spinning Method
Kureha Corporation, Japan	KRECA	1450	Petroleum-based (Isotropic)	Short Fiber	Centrifugal Spinning
Osaka Gas Co., Ltd., Japan	DONACARBO	600	Coal-based (Isotropic)	Crimped Short Fiber	Vortex Spinning
Anshan Senoda Carbon Fiber Co., Ltd., China	/	400	Petroleum-based (Isotropic)	Short Fiber	Melt Blowing
Institute of Coal Chemistry, Chinese Academy of Sciences (CAS)	/	100	Petroleum-based (Isotropic)	Continuous Filament	Melt Spinning
Cytec Engineering Materials, Inc., USA	THORNEL	400	Petroleum-based (Anisotropic)	Continuous Filament	Melt Spinning
Mitsubishi Chemical Corporation, Japan	DIALEAD	1300	Coal-based (Anisotropic)	Continuous Filament	Melt Spinning
Nippon Graphite Fiber Corporation, Japan	GRANOC	180	Coal-based (Anisotropic)	Continuous Filament	Melt Spinning

Table.S2. Common Issues and Solutions in the Pre-oxidation Process of Carbon Fibers

Problem Type	Cause	Mitigation Measures	Detection / Control Methods
Fiber breakage	Rapid oxidation rate leading to localized thermal stress concentration; non-uniform mechanical stretching	Reduce heating rate ; optimize stretching tension	On-line tension sensor monitoring
Non-uniform oxidation	Uneven airflow distribution; excessive fiber tow density	Design multi-stage airflow oxidation furnaces; control tow size	Infrared imaging of C=O distribution
Gas evolution	Instantaneous release of small molecules; insufficient crosslinking	Stepwise isothermal treatment; pre-pressurization	On-line gas analysis using mass spectrometry

Problem Type	Cause	Mitigation Measures	Detection / Control Methods
Over-oxidation	Excessive oxygen partial pressure; local overheating	Dynamically regulate O_2 concentration; apply gradient temperature profile design	Real-time TGA monitoring

Table.S3. Summary of Key Application Fields of Mesophase Pitch-Based Carbon Fibers

Application Field	Specific Applications	Core Advantages
Aerospace	Satellite structural composites (brackets, panels), spacecraft antennas, optical telescope barrels, hypersonic vehicle leading edges	High modulus (excellent resistance to deformation), lightweight, extremely low/negative coefficient of thermal expansion (outstanding dimensional stability), high-temperature resistance
High-end Industry	Semiconductor manufacturing equipment (heaters, thermal shields, wafer holders), precision instruments (robotic arms, measuring rods)	High thermal conductivity, high modulus, low thermal expansion, cleanliness and non-contamination, corrosion resistance
Defense and Military	Radar radomes, stealth materials, lightweight armor, unmanned aerial vehicles (UAVs)	High modulus, good electromagnetic transparency, lightweight, structural–functional integration
Sports and Leisure	Ultra-premium golf clubs, fishing rods, competitive bicycle frames, Formula 1 car components	Exceptional stiffness and resilience (high efficiency in force transfer), lightweight
Scientific Research	Synchrotron radiation sources, particle accelerators (beam pipes, supports), superconducting magnet support structures	High modulus, extremely low coefficient of thermal expansion, stable performance under cryogenic conditions
New Energy & Electronics	Fuel cell bipolar plates, UAV battery packs, heat sinks for high-power electronic devices	High electrical or thermal conductivity, corrosion resistance, lightweight
